# Delivery of selected guideline-based care processes in adult stroke rehabilitation: a systematic review and meta-analysis

**DOI:** 10.3389/fmed.2026.1963143

**Published:** 2026-10-27

**Authors:** Marwan M. A. Aljohani, Abdullah Ibrahim Alhusayni

**Affiliations:** 1 Department of Physical Therapy, College of Medical Rehabilitation Sciences, Taibah University, Madinah, Saudi Arabia; 2 King Salman Center for Disability Research, Riyadh, Saudi Arabia; 3 Department of Health Rehabilitation, College of Applied Medical Sciences at Shaqra, Shaqra University, Shaqra, Saudi Arabia

**Keywords:** clinical practice guidelines, evidence-based practice, guideline adherence, meta-analysis, quality of care, rehabilitation, stroke

## Abstract

**Background:**

Clinical practice guidelines provide evidence-based recommendations for stroke rehabilitation, but the extent to which guideline-concordant care is delivered in routine practice remains uncertain. The goal of this study was to estimate the pooled rate of patient- or chart-level delivery of selected guideline-based care processes in adult stroke rehabilitation.

**Methods:**

A systematic review and meta-analysis was conducted to identify studies reporting patient-, chart-, or care-episode-level adherence to guideline-recommended stroke rehabilitation care. Eligible studies included adults with stroke and reported extractable numerator/denominator data for at least one rehabilitation-relevant guideline-concordant care process.

**Results:**

Six studies were included in the quantitative synthesis, contributing a combined denominator of 547 patients or patient charts. The included studies assessed overall physical therapy adherence, cognitive screening, depression screening, sexuality-related rehabilitation, community rehabilitation contact, and aerobic-intensity therapy. The six studies evaluated distinct care processes in different rehabilitation settings. Individual adherence estimates ranged from 0.0% to 82.9%. The pooled adherence rate was 35.6% (95% CI: 11.0%-71.3%). Heterogeneity was substantial (*τ*^2^ = 3.21; I^2^ = 97.6%; Q(5) = 136.46, *p* < 0.001), showing marked variation in rehabilitation domains, settings, and adherence definitions.

**Conclusion:**

Patient-level adherence to guideline-concordant stroke rehabilitation care appears suboptimal and highly heterogeneous. Given the substantial clinical and construct heterogeneity, the pooled estimate has limited clinical interpretability and primarily provides a descriptive summary of the included care-process measures. These findings highlight persistent evidence-practice gaps in stroke rehabilitation and support the need for targeted implementation strategies, routine audit, and domain-specific quality improvement.

**Systematic Review Registration:**

https://www.crd.york.ac.uk/PROSPERO/view/CRD420261405899, PROSPERO CRD420261405899.

## 1 Introduction

Stroke remains one of the leading global causes of death and long-term disability. Global Burden of Disease 2021 estimates indicate that there were approximately 93.8 million prevalent stroke cases, 11.9 million incident strokes, 7.3 million stroke-related deaths, and 160.5 million disability-adjusted life-years attributable to stroke worldwide in 2021, making stroke a major contributor to neurological and overall disease burden (1). Beyond survival, stroke frequently results in persistent motor impairment, spasticity, dysphagia, cognitive impairment, aphasia, depression, fatigue, reduced independence in activities of daily living, and restrictions in social participation (2). These sequelae often require coordinated rehabilitation among acute, inpatient, outpatient, and community settings (3).

Organized multidisciplinary stroke care and rehabilitation has been associated with improved survival, independence, and likelihood of returning home, supporting the importance of structured rehabilitation pathways (4). Effective stroke rehabilitation is not limited to physical recovery but includes assessment and management of mobility, upper-limb function, communication, swallowing, cognition, mood, fatigue, continence, secondary prevention, discharge planning, caregiver education, and community reintegration (5). Contemporary clinical practice guidelines have been developed to standardize these processes and promote evidence-based care in the post-stroke continuum. For example, contemporary guidelines from the American Heart Association/American Stroke Association (6), the UK National Institute for Health and Care Excellence (NICE) (7), and the Stroke Foundation of Australia (8) provide evidence-based recommendations covering rehabilitation assessment, multidisciplinary therapy, rehabilitation intensity, management of post-stroke impairments, transition of care, and long-term recovery.

Despite the availability of these guidelines, translation into routine rehabilitation practice remains inconsistent. Guideline recommendations may be present at the policy level but incompletely implemented at the bedside, in outpatient clinics, or during community follow-up. Previous patient-level and chart-level studies have shown that adults with stroke do not always receive recommended assessment, screening, therapy intensity, education, or follow-up. For example, adherence has been reported as variable for cognitive screening, depression screening, and physical therapy processes, community rehabilitation contact, aerobic-intensity therapy, and sexuality-related rehabilitation services (9–12). This suggests that the existence of evidence-based recommendations alone is insufficient to ensure delivery of guideline-concordant care.

The evidence-practice gap in stroke rehabilitation is clinically important because many guideline-recommended processes are linked to timely identification and management of potentially modifiable post-stroke impairments and complications (8, 13). Cognitive and mood screening can identify impairments that affect engagement with rehabilitation, safety, discharge planning, caregiver burden, and long-term participation (7, 8). Recommended rehabilitation intensity and timely access to rehabilitation are important components of contemporary stroke rehabilitation pathways and may influence functional recovery and independence. Timely community rehabilitation can support continuity of care after discharge, when patients often face new functional, psychological, and social challenges (7, 14). Therefore, failure to deliver guideline-concordant rehabilitation care may indicate important gaps in care processes and continuity, although the included studies did not directly assess associations with subsequent functional or health outcomes.

Adherence to guideline-recommended rehabilitation care can function as a measurable quality-of-care indicator, particularly when assessed at the patient- or chart-level. In this review, guideline adherence refers to the extent to which delivered or documented care corresponds with recommendations, standards, or measurable quality indicators derived from clinical practice guidelines or best-practice recommendations. A guideline-based quality indicator was considered a measurable care process or performance criterion derived from, or explicitly linked to, a guideline recommendation (15). Patient-level adherence data are especially important because they directly reflect whether an individual patient received the recommended care process. However, the existing literature is fragmented. Many studies focus on single domains, single centres, specific professional groups, or isolated quality indicators. Other studies report service-level structures, clinician attitudes, guideline awareness, or implementation barriers rather than direct patient-level receipt of guideline-concordant rehabilitation care. As a result, the overall magnitude of patient-level adherence to stroke rehabilitation guidelines remains unclear. To address this gap, the present systematic review and meta-analysis aimed to estimate the pooled rate of patient-level adherence to clinical practice guidelines and guideline-based care processes in adult stroke rehabilitation. This study sought to provide a clinically tangible estimate of guideline implementation in routine stroke rehabilitation practice through focusing specifically on patient- or chart-level adherence data.

## 2 Methods

### 2.1 Design of the study

This study was designed as a systematic review and meta-analysis and was conducted in accordance with the Preferred Reporting Items for Systematic Reviews and Meta-Analyses (PRISMA) framework (16). The completed PRISMA 2020 checklist is provided as Supplementary File 1. The protocol of this study was prospectively registered in PROSPERO (registration number: CRD420261405899). The review aimed to synthesize evidence on patient-level adherence to clinical practice guidelines and guideline-based care processes in adult stroke rehabilitation. The quantitative synthesis focused on studies reporting extractable numerator/denominator data for the proportion of patients or patient charts in which guideline-concordant rehabilitation care was delivered or documented.

### 2.2 Eligibility criteria

Studies were considered eligible if they met predefined criteria related to population, setting, outcome, and reporting. Eligible studies included adults with stroke receiving rehabilitation-related care in inpatient rehabilitation, outpatient rehabilitation, community rehabilitation, or rehabilitation-relevant post-acute care settings. Studies were required to assess adherence to clinical practice guidelines, best-practice recommendations, quality indicators, or guideline-based care processes relevant to stroke rehabilitation. Only non-randomized studies were eligible for inclusion.

To be included in the meta-analysis, studies had to report patient- or chart-level numerical data that allowed calculation of an adherence proportion, defined as the number of patients or records receiving guideline-concordant care divided by the total number assessed. Eligible adherence outcomes included, but were not limited to, rehabilitation assessment, physical therapy adherence, cognitive screening, depression screening, rehabilitation service provision, timely community rehabilitation contact, and therapy-intensity targets.

Studies were excluded if they reported only service-level or facility-level indicators, clinician-reported practice, qualitative data, guideline development, implementation barriers without patient-level adherence data, or multiple non-independent item-level indicators without a clearly extractable patient-level adherence outcome. Reviews, editorials, commentaries, protocols, conference abstracts without sufficient data, paediatric studies, and non-English publications were excluded. The review was restricted to English-language publications because resources for reliable translation and assessment of non-English full texts were not available.

### 2.3 Search strategy and study identification

A structured search was performed in PubMed/MEDLINE, Scopus, Web of Science, ProQuest, and Google Scholar (extending from database inception until September 2026) to identify studies evaluating adherence to clinical practice guidelines or guideline-based care processes in adult stroke rehabilitation. Since Google Scholar returns wide-ranging results ordered by relevance, screening was limited to the first 200 records, an approach endorsed in the systematic review methodology literature as a way of balancing comprehensiveness with feasibility (17). The search strategy combined terms and keywords including stroke rehabilitation, clinical practice guidelines, guideline adherence, evidence-based practice, best practice, quality of care, audit, chart review, medical record review, and patient-level care processes. Boolean operators (such as AND and OR) were used to combine relevant concepts and to improve retrieval of studies reporting rehabilitation-related adherence outcomes. The complete database-specific electronic search strategies, including controlled vocabulary where applicable, free-text terms, Boolean operators, and applied limits, are provided in the Supplementary File 2. It should be noted that grey literature sources, dissertations, theses, trial registries, and non-indexed reports were not systematically searched. However, the reference lists of included studies were also manually screened to find potential eligible reports.

The search was focused on patient-level stroke rehabilitation adherence rather than broader neurological rehabilitation or service-level implementation. Records retrieved from the search were screened against the eligibility criteria. Additional potentially relevant studies identified during literature searching and full-text screening were also considered if they reported extractable patient- or chart-level data permitting calculation of the proportion receiving or documenting a guideline-concordant rehabilitation care process, consistent with the prespecified eligibility criteria. Duplicate records and duplicate reports of the same dataset were removed before final inclusion.

### 2.4 Screening of studies

Title and abstract screening was conducted to identify studies potentially relevant to adult stroke rehabilitation guideline adherence. Records that were clearly irrelevant, non-stroke, paediatric, non-rehabilitation-focused, qualitative-only, review articles, or service-level reports without patient-level adherence data were excluded at this stage.

Full texts were retrieved for all studies considered potentially eligible. During full-text assessment, studies were evaluated according to whether they reported extractable patient- or chart-level adherence data. Particular attention was given to whether the denominator represented individual patients or records rather than hospitals, clinicians, facilities, or guideline items. Studies that reported multiple process indicators without one clearly prespecified patient-level adherence outcome were excluded from the meta-analysis. Titles and abstracts were independently screened by two reviewers (M.M.A.A. and A.I.A.), followed by independent full-text assessment of potentially eligible studies. Disagreements or uncertainties were resolved through discussion and consensus between the two reviewers.

### 2.5 Data extraction

Data were independently extracted by two reviewers (M.M.A.A. and A.I.A.) using a standardized extraction form developed for this review, with discrepancies resolved through discussion and consensus. Extracted study characteristics included first author, year of publication, country, study design, data source, population, rehabilitation setting, sample size, guideline or recommendation assessed, adherence domain, and the specific adherence outcome used for quantitative synthesis. We also recorded whether each outcome reflected documented care, audited service delivery, or direct measurement of care delivery.

For the meta-analysis, extraction focused on binary patient-level adherence data. The numerator was defined as the number of patients, charts, records, or care episodes in which the guideline-concordant care process was delivered. The denominator was defined as the total number of eligible patients, charts, records, or care episodes assessed. Where studies reported percentages and total sample size but not exact event counts, event numbers were calculated when appropriate and clearly noted. In before-after implementation studies, the baseline or pre-intervention routine-care timepoint was selected for the primary meta-analysis to avoid mixing usual-care adherence with post-intervention effects. Where multiple related publications addressed the same rehabilitation domain, the publication providing the most directly extractable patient-level numerator/denominator data corresponding to the predefined outcome was selected for quantitative synthesis. Accordingly, Auger *et al*. (2025) (18) was included because it reported an explicit baseline patient-level estimate (5/15 patients) for provision of sexuality-related rehabilitation services, whereas the earlier Auger et al. (10) study (2022) did not provide the same eligible patient-level adherence outcome.

### 2.6 Risk-of-bias assessment

Risk of bias and methodological quality of the included studies were assessed using the Joanna Briggs Institute (JBI) Critical Appraisal Checklist for Studies Reporting Prevalence Data (19). This tool was selected as the quantitative synthesis was based on study-level proportions of patients, charts, records, or care episodes receiving guideline-concordant rehabilitation care, despite variation in the nominal study designs. The checklist assesses the appropriateness of the sampling frame and sampling method, adequacy of sample size, description of participants and setting, coverage of the identified sample, validity and consistency of outcome ascertainment, appropriateness of statistical analysis, and adequacy of response or data completeness. Each item was categorized as “Yes,” “No,” “Unclear,” or “Not applicable.” No numerical summary score was calculated, and studies were not excluded on the basis of the appraisal; instead, identified methodological limitations were considered when interpreting the pooled findings. Two reviewers (M.M.A.A. and A.I.A.) independently assessed each study, with disagreements resolved through discussion and consensus. Detailed assessments are provided in Supplementary File 3.

### 2.7 Primary outcome and adherence definition

For consistency, outcomes were described as patient- or chart-level. Patient-level outcomes used individual patients as the denominator, whereas chart-level outcomes were derived from individual patient medical records. These were analysed together because both reflected delivery or documentation of guideline-based care at the individual-patient level.

The primary outcome was the pooled proportion of patient- or chart-level delivery of selected guideline-based rehabilitation care processes. For this review, guideline-concordant care was defined as documented delivery of a rehabilitation-related assessment, intervention, service, or care process that was consistent with a specific recommendation, target, protocol, pathway, or quality indicator derived from an established clinical practice guideline or best-practice standard. Concordance was assessed according to the operational definition used in each included study and did not require adherence to every recommendation within an entire guideline (15). The unit of analysis was the patient, chart, record, or care episode. The adherence proportion was calculated as: Adherence proportion = number receiving guideline-concordant care​/total number assessed.

The included care-process domains were not selected *a priori*. Rather, they reflected the rehabilitation-related guideline-concordant outcomes reported by studies that met the predefined eligibility criteria, including physical therapy adherence, cognitive screening, depression screening, sexuality-related rehabilitation, community rehabilitation contact within a recommended timeframe, and achievement of recommended aerobic therapy intensity. Accordingly, the domains represented the available eligible evidence rather than a prespecified framework of rehabilitation domains. The source, version, recommendation characteristics, applicability, and operational definition of each included guideline-based outcome are detailed in Supplementary File 4. Because these domains represent different components of stroke rehabilitation, the pooled estimate was interpreted as a broad implementation-performance indicator rather than a measure of adherence to a single homogeneous recommendation.

### 2.8 Statistical methods

The meta-analysis was performed using patient-, chart-, or care-episode-level adherence proportions. For each included study, the number of adherent cases and the total number assessed were extracted. Adherence percentages were calculated for descriptive reporting, but the meta-analysis was based on raw numerator and denominator data. Because the outcome was a single-group proportion and one study reported zero events, proportions were transformed using a logit transformation with continuity correction where required (20). A continuity correction of 0.5 was added to the event and non-event counts of all included studies before calculating the logit-transformed proportions and corresponding standard errors. Random-effects meta-analysis was used because substantial clinical and methodological heterogeneity was expected in rehabilitation settings, adherence domains, and guideline recommendations. The random-effects model was estimated using restricted maximum likelihood (REML) (21). Pooled estimates were back-transformed from the logit scale and reported as adherence percentages with corresponding 95% confidence intervals. Between-study heterogeneity was assessed using Cochran's Q test, with a *p* value below 0.10 considered indicative of statistically significant heterogeneity (22). Heterogeneity was further quantified using the I^2^ statistic, with approximate values of 25%, 50%, and 75% interpreted as low, moderate, and high heterogeneity, respectively. The between-study variance (*τ*^2^) and H^2^ statistic were also reported. Leave-one-out sensitivity analysis was performed by removing each study in turn and repeating the meta-analysis to evaluate whether the pooled estimate was disproportionately influenced by any single study. Galbraith plots were used as an exploratory visual assessment of between-study heterogeneity (23). Funnel plots were generated to explore small-study effects (24). All statistical analyses were performed using Stata version 17 (StataCorp, College Station, TX, USA).

## 3 Results

### 3.1 Study selection and characteristics

A total of 923 records were identified through database searching, including PubMed, Scopus, Web of Science, ProQuest, and Google Scholar. After removal of duplicate records (*n* = 445), 478 records remained for title and abstract screening. Of these, 358 records were excluded as irrelevant or not meeting the review scope. The full texts of 120 articles were assessed for eligibility, of which 114 were excluded: 26 were review articles, 64 had unsuitable or non-extractable data, and 24 reported irrelevant outcomes or populations. Finally, six studies met the inclusion criteria and were included in the quantitative meta-analysis. The process of literature search and study selection is depicted in the flow-diagram Figure 1.

**Figure 1:**
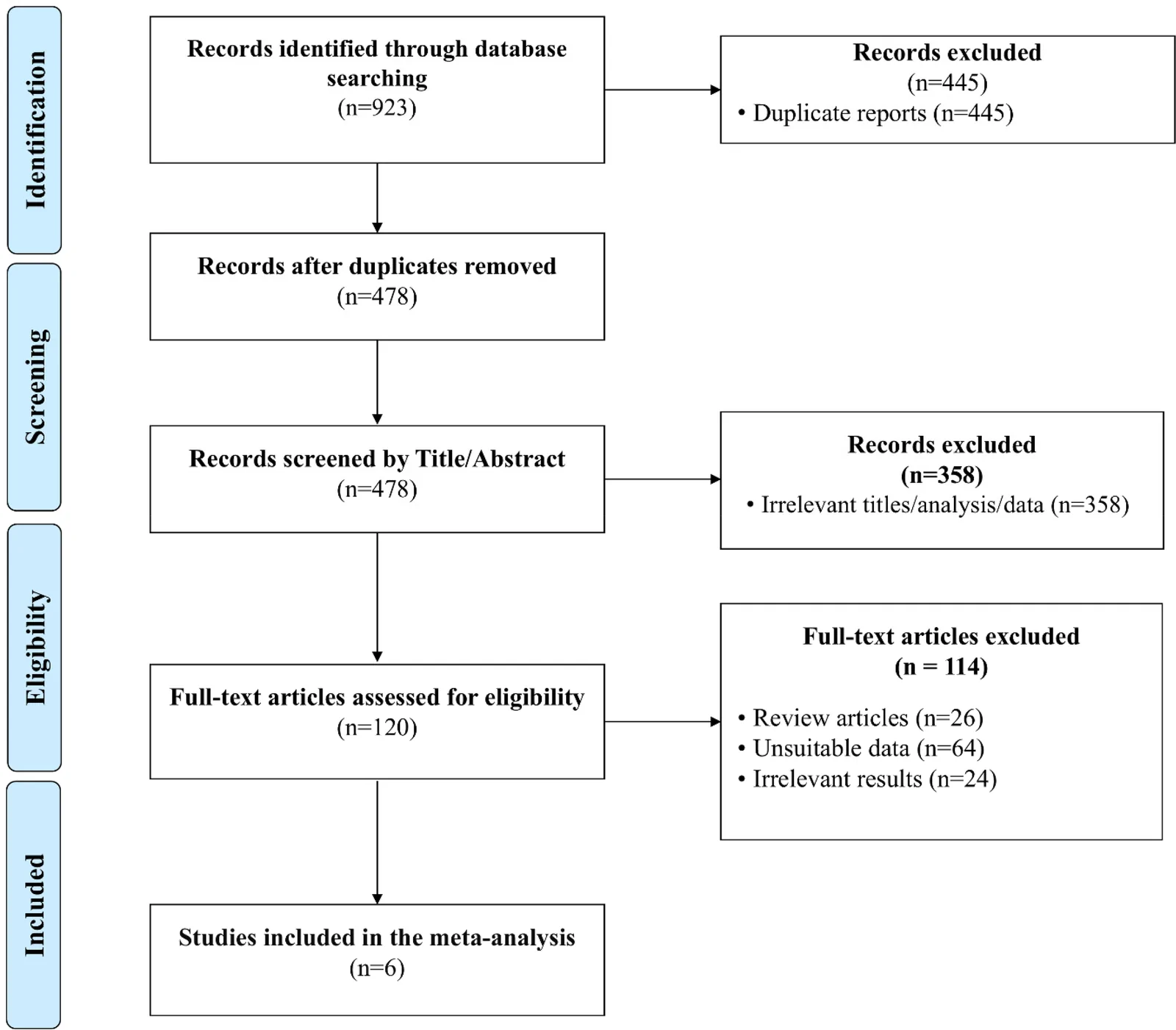
Flow diagram of literature search and screening based on PRISMA.

Six studies met the eligibility criteria for the primary patient-/chart-level quantitative synthesis, including Ajimsha *et al*., 2019 (9), Auger *et al*., 2025 (18), McClure *et al*., 2012 (11), MacKenzie *et al*., 2019 (12), Thompson *et al*., 2019 (25), Yan *et al*., 2025 (26) (Table 1). All included studies focused on adult stroke rehabilitation or rehabilitation-relevant care processes. The studies were conducted in Qatar, Canada, and New Zealand, and included inpatient rehabilitation, outpatient rehabilitation, and community rehabilitation, where the assessed process was directly related to rehabilitation care. The included studies assessed different guideline-concordant care processes, including overall physical therapy adherence, cognitive screening, depression screening, sexuality-related rehabilitation, timely community rehabilitation review, and achievement of recommended aerobic therapy intensity.

**Table 1:** Specifications of the studies included in the study.

Study	Country	Design/data source	Data collection method	Outcome ascertainment	Population and setting	Sample/unit included in meta-analysis	Guideline/recommendation assessed	Adherence outcome used for meta-analysis	Event/total (unit)	Adherence (%)	Timepoint used
Ajimsha *et al*., 2019 (9)	Qatar	Retrospective chart audit	Retrospective medical-record/chart audit	Documentation-based care assessment from patient charts	Adults with stroke admitted to an active inpatient stroke rehabilitation centre; physical therapy records audited against the PAAS guideline	127 patient charts	Physical Therapy After Acute Stroke guideline	Overall physical therapy adherence to the structured stroke rehabilitation guideline	90/127 charts	70.9	Routine care
Auger *et al*., 2025 (18)	Canada	Pilot implementation study with monthly patient audits	Monthly patient audits during implementation study	Service delivery assessed through structured monthly patient audits	Adults receiving inpatient stroke rehabilitation in one rehabilitation centre	15 patients	Stroke rehabilitation recommendations addressing sexuality-related rehabilitation	Sexuality addressed or sexuality-related rehabilitation service provided during inpatient rehabilitation	5/15 patients	33.3	Baseline/routine care
McClure *et al*., 2012 (11)	Canada	Retrospective chart review	Retrospective medical-record review	Documentation-based assessment from patient charts	Adults admitted to an inpatient stroke rehabilitation facility	123 patients	Canadian Best Practice Recommendations for Stroke Care; vascular cognitive impairment screening/assessment recommendation	Documented completion of a formal cognitive screening instrument (MoCA or MMSE) during inpatient rehabilitation; modified or incomplete versions were not considered complete formal screens	102/123 patients	82.9	Routine care
MacKenzie *et al*., 2019 (12)	Canada	Retrospective before-after chart review	Retrospective chart review	Documentation-based assessment from patient charts	Adults attending an outpatient stroke rehabilitation clinic after discharge	70 patient charts	Canadian Best Practice Recommendations for depression screening	Depression screening using a standardized screening tool	0/70 charts	0.0	Pre-intervention/routine care
Thompson *et al*., 2019 (25)	New Zealand	Prospective service audit	Prospective service audit	Actual service delivery captured through prospective service audit	Consecutive adults with new stroke referred to a community rehabilitation team in Wellington	46 patients with complete timing data	New Zealand Stroke Network community rehabilitation recommendations/Ministry of Health community rehabilitation indicator	Face-to-face community rehabilitation contact within seven calendar days after hospital discharge	17/46 patients	37.0	Routine care
Yan *et al*., 2025 (26)	Canada	Cross-sectional analysis of usual-care arm data	Direct monitoring/measurement during a physical therapy session	Direct objective measurement during a monitored physical therapy session	Medically stable adults admitted for inpatient stroke rehabilitation across Canadian sites; physical therapy session monitored at rehabilitation midpoint	166 participants	Guideline target for aerobic intensity during stroke rehabilitation: ≥20 min at aerobic intensity during a session	Achieved ≥20 aerobic minutes during the monitored physical therapy session	30/166 participants	18.1	Usual care

The combined denominator in the six included studies was 547 patients or patient charts, with individual study sample sizes ranging from 15 to 166. Adherence estimates varied markedly among studies, from 0.0% for routine depression screening before implementation of a standardized form to 82.9% for cognitive screening during inpatient stroke rehabilitation.

### 3.2 Risk-of-bias assessment

The methodological appraisal identified several study-specific limitations (Supplementary File 3). The most common concerns related to restricted single-centre sampling, reliance on retrospective chart documentation, incomplete or selectively available records, and limited sample size. Ajimsha *et al*. (9) included 127 of 216 initially identified records, raising concern regarding coverage of the eligible sample, whereas the baseline estimate from Auger *et al*. (18) was based on only 15 patients. Thompson *et al*. (25) used consecutive prospective sampling, although complete data for the timing indicator were available for 46 of 50 participants. McClure *et al*. (11) and MacKenzie *et al*. (12) relied on retrospective documentation of screening processes. Yan et al. (26) used objective measurement across multiple rehabilitation sites, but the adherence estimate represented achievement of the aerobic-intensity target during a single monitored physical-therapy session rather than across the rehabilitation episode. Therefore, these limitations were considered when interpreting the pooled estimate and contributed to the need for cautious interpretation.

### 3.3 Pooled meta-analysis

The primary random-effects meta-analysis included six patient-/chart-level adherence estimates. Using a REML random-effects model with logit-transformed proportions, the pooled logit estimate was −0.59 with a 95% confidence interval of −2.09 to 0.91. After back-transformation, the pooled adherence rate was approximately 35.6% (95% CI: 11.0%-71.3%) (Figure 2).

**Figure 2:**
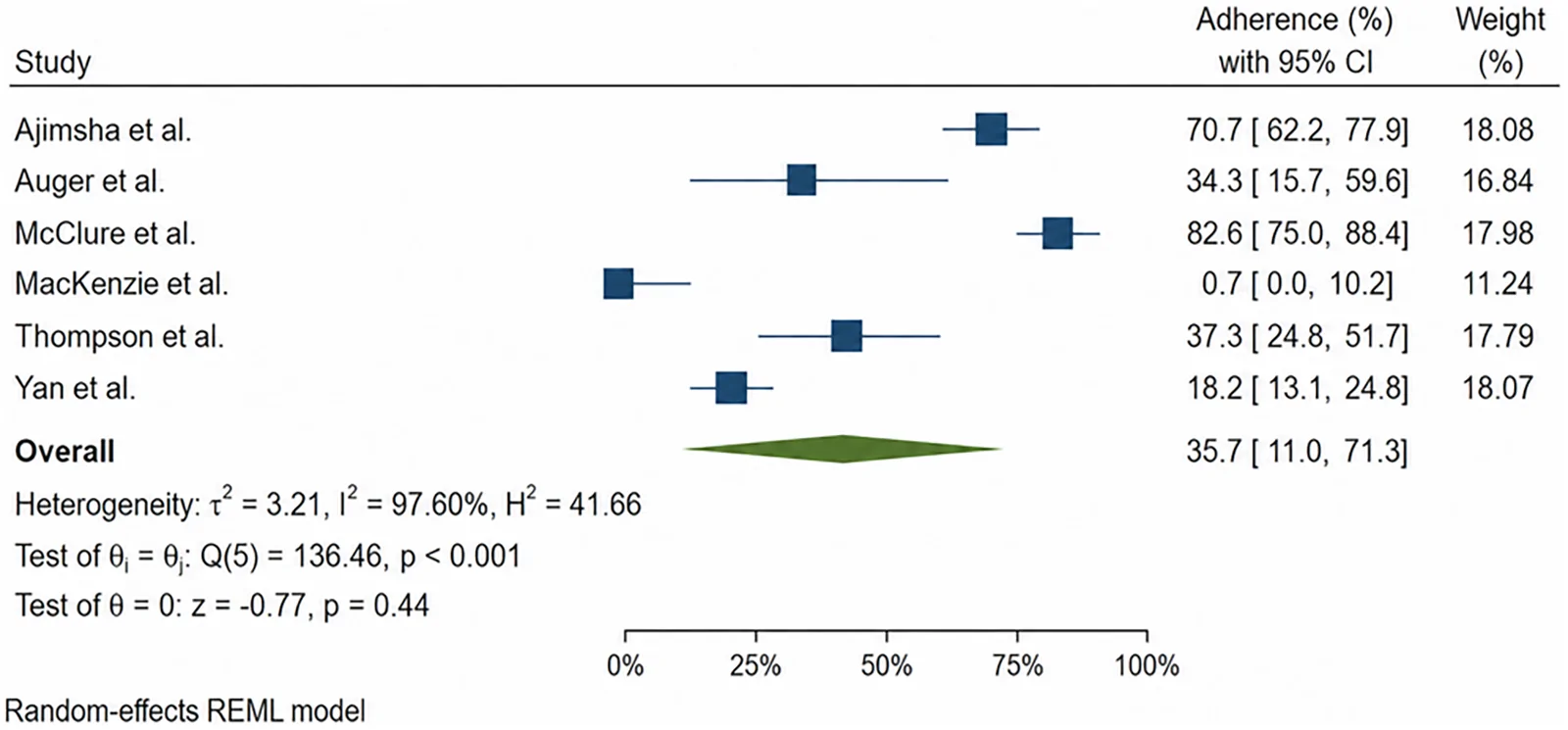
Forest plot of patient-level adherence to guideline-concordant adult stroke rehabilitation care. Random-effects meta-analysis of six patient-/chart-level studies reporting adherence to stroke rehabilitation clinical practice guidelines or guideline-based care processes. The meta-analysis was conducted using logit-transformed proportions, with study estimates and the pooled estimate displayed as back-transformed percentages. The pooled logit estimate was −0.59 (95% CI: −2.09 to 0.91), corresponding to a back-transformed pooled adherence rate of approximately 35.6% (95% CI: 11.0%–71.3%). Heterogeneity was substantial (*τ*^2^ = 3.21; I^2^ = 97.6%; Q(5) = 136.46, *p* < 0.001).

### 3.4 Individual study adherence estimates

The highest adherence estimate was reported by McClure *et al*. (11), where 102 of 123 patients received formal cognitive screening during inpatient stroke rehabilitation, corresponding to an adherence rate of 82.9%. Ajimsha *et al*. (9), reported overall physical therapy guideline adherence of approximately 70.9% based on 90 of 127 charts.

Lower adherence was observed in studies assessing more specific or less routinely embedded rehabilitation processes. Auger *et al*. (18) reported that sexuality-related rehabilitation services were provided to 5 of 15 patients at baseline (33.3%). Thompson *et al*. (25). reported timely community rehabilitation contact within seven calendar days for 17 of 46 patients (37.0%). Yan *et al*. (26) reported that 30 of 166 patients achieved at least 20 min of aerobic-intensity activity during a monitored inpatient rehabilitation session (18.1%). The lowest estimate was from MacKenzie *et al*. (12) where routine depression screening before implementation of a standardized form was 0 of 70 charts (0.0%).

### 3.5 Heterogeneity

Substantial between-study heterogeneity was observed (*τ*^2^ = 3.21; I^2^ = 97.6%; H^2^ = 41.66). The test for heterogeneity was statistically significant (Q(5) = 136.46, *p* < 0.001), indicating that the included studies were not estimating a single uniform adherence construct (Figure 2). This heterogeneity is consistent with the clinical and methodological diversity of the included adherence indicators, which covered different domains of stroke rehabilitation care. Given this clinical and construct heterogeneity, the pooled estimate should not be interpreted as a common underlying effect across studies. Statistical pooling is presented only as a descriptive summary of the observed proportions, and its appropriateness is limited when the included estimates represent different rehabilitation constructs.

### 3.6 Small-study effects and outlier assessment

Visual inspection of the funnel plot suggested asymmetry (Figure 3); however, this should be interpreted cautiously because only six studies were included. With such a number of studies, funnel plots and formal publication-bias assessments have limited reliability. The exploratory Galbraith plot (Figure 4) showed substantial dispersion of study estimates, consistent with the marked clinical and methodological heterogeneity among the included studies.

**Figure 3:**
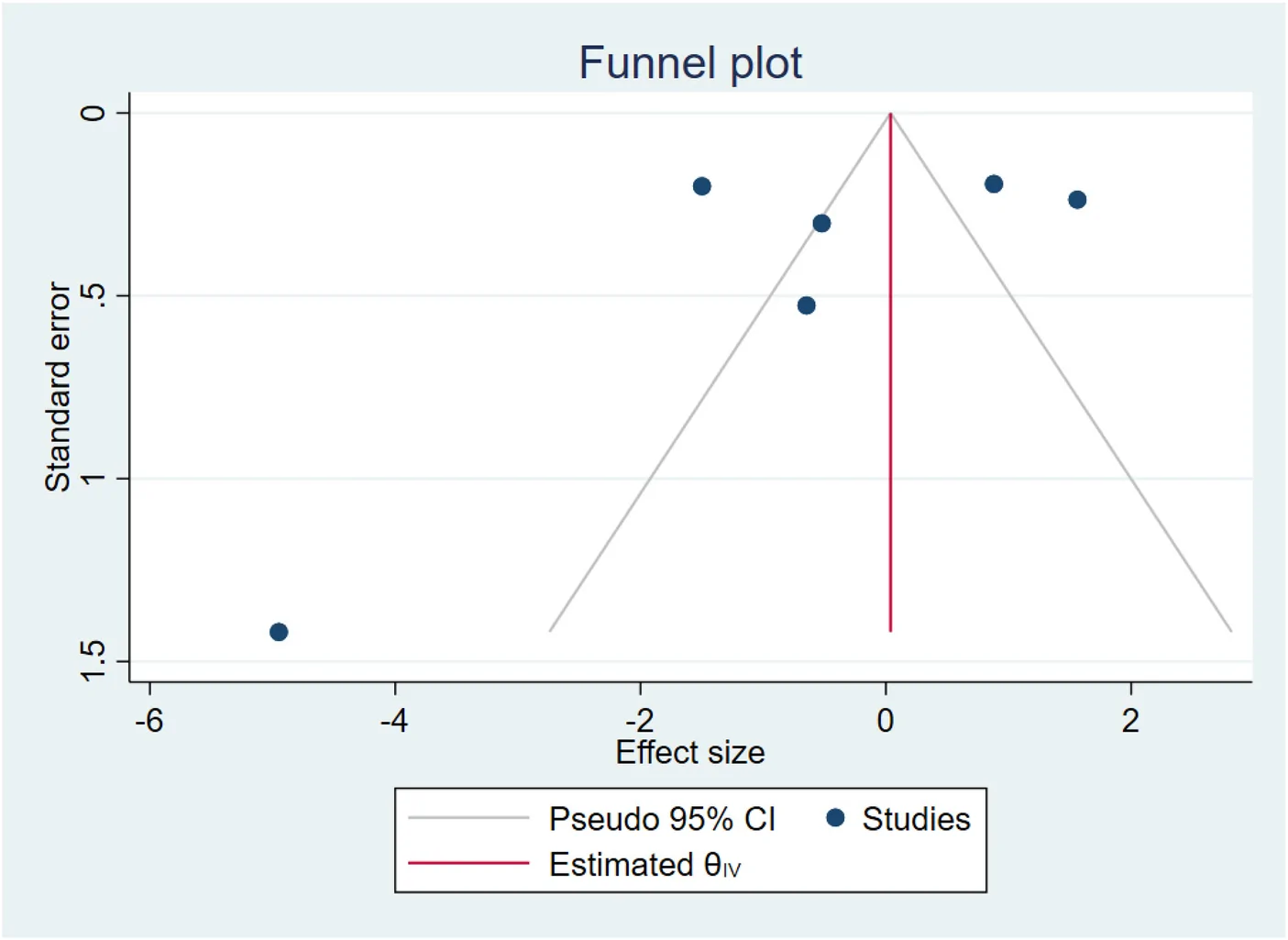
Funnel plot of included studies. Funnel plot assessing small-study effects among studies included in the primary meta-analysis. Visual asymmetry should be interpreted cautiously because only six studies were included, and funnel plot interpretation is unreliable with fewer than 10 studies.

**Figure 4:**
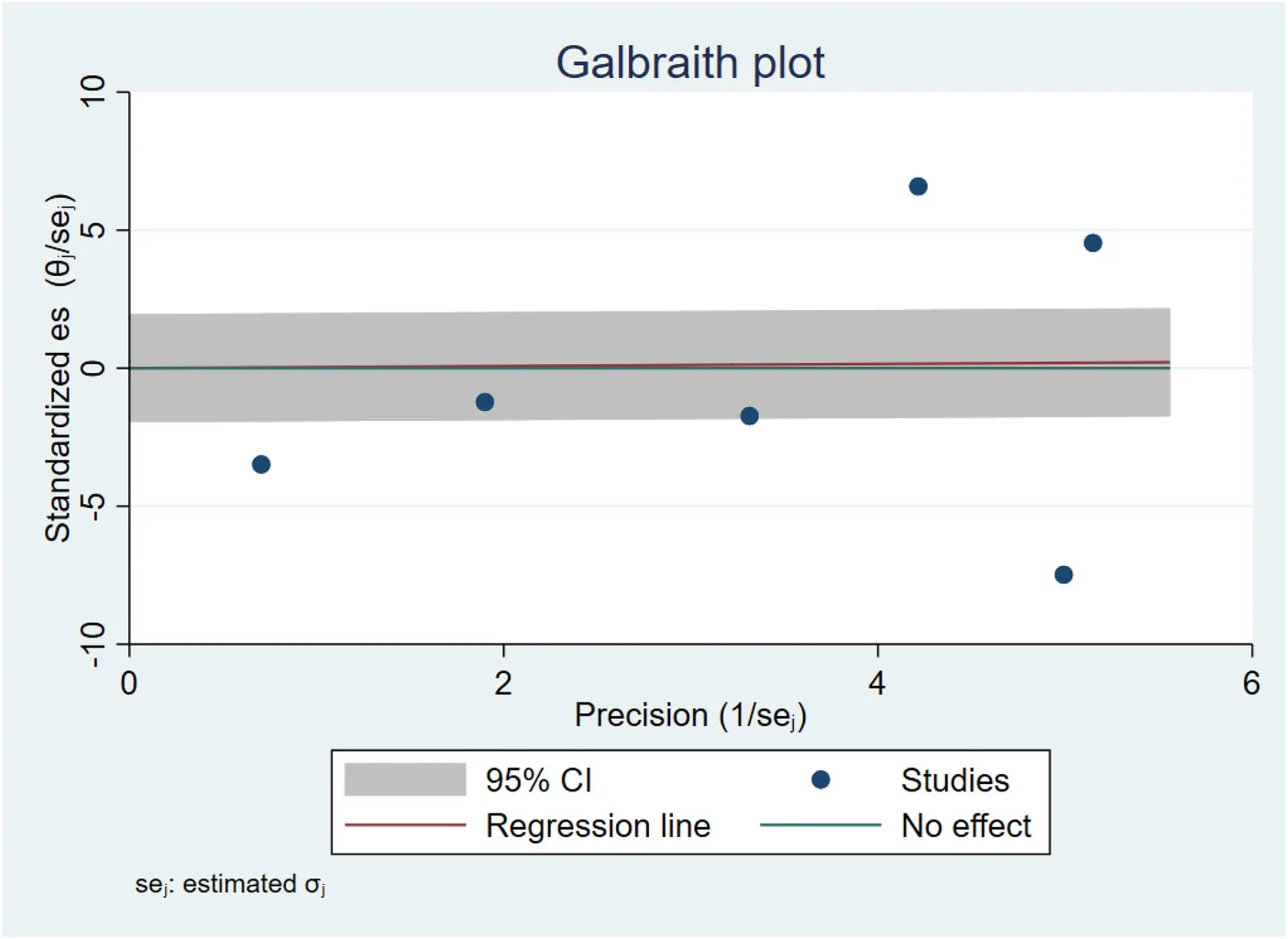
Galbraith plot for heterogeneity assessment. Galbraith plot showing study-level contributions to heterogeneity in the logit-transformed adherence analysis. Several studies fell outside the expected limits, supporting substantial between-study heterogeneity and indicating that variation in rehabilitation domain, setting, and adherence definition contributed to dispersion of effect estimates.

### 3.7 Sensitivity analysis

Leave-one-out sensitivity analysis showed that the pooled estimate remained imprecise regardless of which study was omitted (Figure 5). The back-transformed pooled adherence estimates ranged from approximately 26.7% when McClure *et al*. was omitted to 49.3% when MacKenzie *et al*. was omitted. Omission of MacKenzie *et al*., the study with zero baseline screening events, produced the largest upward shift in the pooled estimate, suggesting that this study contributed substantially to the low overall pooled adherence estimate. However, all leave-one-out confidence intervals remained wide, indicating persistent uncertainty due to the small number of studies and high heterogeneity.

**Figure 5:**
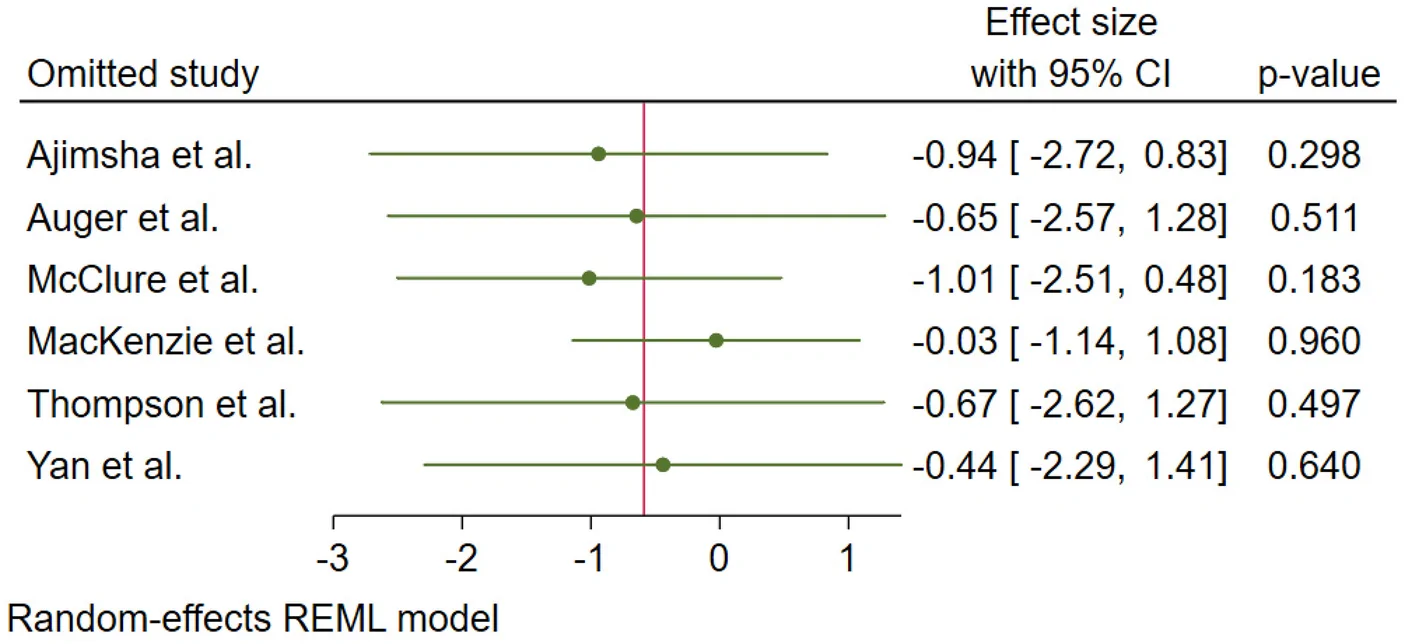
Leave-one-out sensitivity analysis. Sensitivity analysis evaluating the influence of each included study on the pooled adherence estimate. The pooled estimate remained imprecise after omission of individual studies, indicating that uncertainty persisted in analyses. Exclusion of the zero-event depression-screening study produced the largest upward shift in the pooled adherence estimate.

## 4 Discussion

This systematic review and meta-analysis found substantial variation in patient- or chart-level delivery of selected guideline-based rehabilitation care processes. In six patient- or chart-level studies, the pooled adherence rate was approximately 35.6% (95% CI: 11.0%–71.3%). However, heterogeneity was substantial, indicating that the included studies were not estimating one uniform care process. Rather, they captured different rehabilitation domains, including physical therapy adherence, cognitive screening, depression screening, sexuality-related rehabilitation, timely community rehabilitation contact, and aerobic-intensity therapy (9, 11, 12, 18, 25, 26). Therefore, the pooled estimate should be interpreted cautiously as a descriptive summary of the included studies rather than as a common measure of rehabilitation quality or guideline adherence. Accordingly, the pooled value should not be interpreted as a single common adherence effect, and its clinical meaning is constrained by the substantial construct heterogeneity in the included studies. It should also be noted that the present analysis was limited to care-process adherence and did not evaluate associations with subsequent functional or recovery outcomes.

To our knowledge, this is the first meta-analysis to quantitatively pool patient- or chart-level adherence rates to guideline-concordant care processes in adult stroke rehabilitation. Previous systematic reviews have mainly focused on the quality of stroke rehabilitation guidelines, clinician awareness, implementation barriers and facilitators, or narrative evidence-practice gaps. For example, Cormican *et al*. systematically reviewed healthcare professionals’ perceived barriers and facilitators to implementing stroke rehabilitation clinical practice guidelines, but did not pool patient-level adherence rates (27). The present review extends this literature by asking a more direct clinical question, whether adults with stroke actually received guideline-concordant rehabilitation care.

The findings are also consistent with recent stroke rehabilitation literature showing that implementation of clinical practice guidelines is influenced by clinician knowledge and skills, competing priorities, available resources, organizational processes, and local support (27, 28). In the included studies, adherence was relatively higher for some established processes, such as cognitive screening and structured physical therapy adherence, but lower for less routinely embedded domains, including depression screening, sexuality-related rehabilitation, timely community follow-up, and aerobic-intensity therapy (9, 11, 12, 18, 25, 26). These differences may reflect variation in the implementation demands of different rehabilitation processes. However, this hypothesis should be examined directly in future studies.

Several implementation barriers may contribute to the low and variable adherence observed, including limited guideline knowledge or confidence, competing clinical priorities, unclear professional roles, and organizational or resource constraints (29, 30). These factors have been reported in stroke-rehabilitation implementation literature, although they were not directly assessed in all studies included in the present meta-analysis. At the organizational level, limited staffing, restricted therapy time, poor integration of guideline prompts into documentation systems, insufficient audit-feedback mechanisms, and variable access to multidisciplinary services may prevent consistent delivery of guideline-concordant care (31). These barriers align with previous stroke rehabilitation implementation evidence, where organizational constraints, limited resources, lack of knowledge and skills, and concerns about guideline applicability have been repeatedly identified as barriers (27).

Based on previous implementation literature, potential strategies for future quality-improvement initiatives include embedding guideline recommendations into routine clinical forms, electronic medical-record prompts, structured assessment templates, audit and feedback, staff education, local clinical champions, multidisciplinary team meetings, clear referral pathways, and performance monitoring (32). Cormican et al. similarly identified training, local protocols, performance monitoring, and leadership as perceived facilitators of guideline implementation in stroke rehabilitation (27). Importantly, implementation strategies should be personalized to the target care process. Improving depression or cognitive screening may require standardised tools and documentation prompts, whereas improving therapy intensity may require workforce planning, scheduling redesign, and greater rehabilitation resources (31).

Although none of the included studies were conducted in Saudi Arabia or other Arab countries, the findings may have contextual relevance for health systems evaluating the implementation of stroke rehabilitation recommendations. Previous literature from Saudi Arabia and the wider Arab region has identified challenges related to access, service organization, and delivery of stroke rehabilitation care (33–35). However, the generalizability of the present findings to these settings remains uncertain. Accordingly, the results should primarily be considered as supporting the need for region-specific audit and implementation studies to evaluate the delivery of guideline-recommended rehabilitation processes within local healthcare systems.

This review has several strengths. It focused on patient-, chart-, or care-episode-level adherence rather than service-level availability or clinician-reported practice, making the findings more directly relevant to patient care. It also used a conservative inclusion approach to avoid combining patient-level estimates with facility-level surveys, qualitative studies, or non-independent item-level indicators. This improves interpretability and reduces the risk of overstating guideline implementation based on indirect measures. On the other hand, several limitations should also be acknowledged. First, the number of included studies was small due to strict patient-level inclusion criteria, limiting precision and preventing robust subgroup analyses by rehabilitation domain, setting, or country. In addition, the review was restricted to non-randomized studies in accordance with the prespecified protocol, which may have limited the breadth of the available evidence. Second, the included studies assessed different guideline-concordant care processes, contributing to substantial heterogeneity. Third, the risk-of-bias assessment identified limitations including single-centre sampling, retrospective chart-based ascertainment, incomplete data, and small sample sizes. These factors may have influenced individual estimates and warrant cautious interpretation of the pooled result.

## 5 Conclusion

Overall, this meta-analysis identified substantial variation in the delivery of guideline-concordant rehabilitation care processes across the included studies. The pooled estimate of approximately 36% represents a descriptive summary of heterogeneous care-process measures and has limited interpretability as an overall measure of patient-level guideline adherence or the magnitude of the evidence-practice gap. The wide variation across studies highlights that adherence is strongly influenced by the specific rehabilitation domain, care setting, documentation system, and resources available to support implementation. These findings support the need for routine patient-level audit of stroke rehabilitation care, rather than relying only on the presence of guidelines, protocols, or service-level structures. Rehabilitation services should identify whether patients actually receive key recommended processes, including screening, assessment, therapy delivery, education, discharge planning, and follow-up. Targeted implementation strategies, such as standardized documentation templates, electronic prompts, audit and feedback, staff education, local clinical champions, and multidisciplinary referral pathways, may help improve delivery of evidence-based stroke rehabilitation care.

## Data Availability

The original contributions presented in the study are included in the article/Supplementary Material, further inquiries can be directed to the corresponding author.
