# Supplementary material for: Delivery of selected guideline-based care processes in adult stroke rehabilitation: a systematic review and meta-analysis
Source: Front Med (Lausanne). 2026 Oct 27;13:1963143. doi: 10.3389/fmed.2026.1963143 (PMC13747950; doi:10.3389/fmed.2026.1963143)
Supplement: Supplementary file 2 [file Table2.docx]

**Supplementary File 2.** Syntax used for literature search in databases.

**Pubmed (n= 184)**

("stroke rehabilitation"[tiab] OR "post-stroke rehabilitation"[tiab] OR "poststroke rehabilitation"[tiab] OR "Stroke Rehabilitation"[Mesh] ) AND ( guideline*[tiab] OR "clinical practice guideline*"[tiab] OR "best practice"[tiab] OR "evidence-based practice"[tiab] OR "Guideline Adherence"[Mesh] ) AND ( adheren*[tiab] OR complian*[tiab] OR concordan*[tiab] OR audit*[tiab] OR "chart review*"[tiab] OR "medical record review*"[tiab] OR "quality of care"[tiab] OR "process of care"[tiab] OR "processes of care"[tiab] ) AND ( patient*[tiab] OR inpatient*[tiab] OR outpatient*[tiab] OR "medical record*"[tiab] OR chart*[tiab] OR file*[tiab] OR record*[tiab] )

**Scopus (n=265)**

TITLE-ABS-KEY ("stroke rehabilitation" OR "post-stroke rehabilitation" OR "poststroke rehabilitation") AND TITLE-ABS-KEY (guideline* OR "clinical practice guideline*" OR best practice" OR "evidence-based practice") AND TITLE-ABS-KEY (adheren* OR complian* OR concordan* OR audit* OR "chart review*" OR "medical record review*" OR "quality of care" OR "process of care" OR "processes of care") AND TITLE-ABS-KEY ( patient* OR inpatient* OR outpatient* OR "medical record*" OR chart* OR file* OR record*)

**ISI Web of Science (n=145)**

TS=("stroke rehabilitation" OR "post-stroke rehabilitation" OR "poststroke rehabilitation") AND TS=(guideline* OR "clinical practice guideline*" OR "best practice" OR "evidence-based practice") AND TS=(adheren* OR complian* OR concordan* OR audit* OR "chart review*" OR "medical record review*" OR "quality of care" OR "process of care" OR "processes of care") AND TS=(patient* OR inpatient* OR outpatient* OR "medical record*" OR chart* OR file* OR record*)

**ProQuest (n=129)**

ABSTRACT,TITLE("stroke rehabilitation" OR "post-stroke rehabilitation" OR "poststroke rehabilitation") AND ABSTRACT,TITLE(guideline* OR "clinical practice guideline" OR "clinical practice guidelines" OR "best practice" OR "evidence-based practice") AND ABSTRACT,TITLE(adheren* OR complian* OR concordan* OR audit* OR "chart review" OR "chart reviews" OR "medical record review" OR "medical record reviews" OR "quality of care" OR "process of care" OR "processes of care") AND ABSTRACT,TITLE(patient* OR inpatient* OR outpatient* OR "medical record" OR "medical records" OR chart* OR file* OR record*)

**Google scholar (n=200)**

("stroke rehabilitation" OR "post-stroke rehabilitation" OR "poststroke rehabilitation") (guideline OR guidelines OR "clinical practice guideline" OR "clinical practice guidelines" OR "best practice" OR "evidence-based practice") (adherence OR compliance OR concordance OR audit OR "chart review" OR "chart reviews" OR "medical record review" OR "medical record reviews" OR "quality of care" OR "process of care" OR "processes of care") (patient OR patients OR inpatient OR inpatients OR outpatient OR outpatients OR "medical record" OR "medical records" OR chart OR charts OR file OR files OR record OR records)
