# Supplementary material for: Delivery of selected guideline-based care processes in adult stroke rehabilitation: a systematic review and meta-analysis
Source: Front Med (Lausanne). 2026 Oct 27;13:1963143. doi: 10.3389/fmed.2026.1963143 (PMC13747950; doi:10.3389/fmed.2026.1963143)
Supplement: Supplementary file 3 [file Table3.docx]

**Supplementary File 3**. Critical appraisal of included studies using the JBI Critical Appraisal Checklist for Studies Reporting Prevalence Data.

| **Study** | **Q1 Sample frame** | **Q2 Sampling** | **Q3 Sample size** | **Q4 Subjects/setting described** | **Q5 Adequate coverage** | **Q6 Valid outcome identification** | **Q7 Standard/reliable measurement** | **Q8 Appropriate analysis** | **Q9 Response/completeness** | **Main methodological considerations** |
| --- | --- | --- | --- | --- | --- | --- | --- | --- | --- | --- |
| Ajimsha *et al*., 2019  (1) | Yes | Unclear | Yes | Yes | No | Yes | Yes | Yes | No | Retrospective single-centre chart audit. Of 216 identified case files, only 127 were included; therefore incomplete coverage/record eligibility may introduce selection bias. |
| Auger *et al*., 2025  (2) | Yes | Unclear | No | Yes | Unclear | Yes | Yes | Yes | Unclear | Single-centre pilot implementation study; only 15 patients contributed to the baseline estimate, producing substantial imprecision and limited representativeness. |
| McClure *et al*., 2012  (3) | Yes | Yes | Yes | Yes | Yes | Yes | Yes | Yes | Yes | Single inpatient rehabilitation facility and retrospective chart ascertainment; documented screening may underestimate undocumented care and does not establish whether screening was appropriately acted upon. |
| MacKenzie *et al*., 2019  (4) | Yes | Unclear | Yes | Yes | Yes | Yes | Yes | Yes | Yes | Retrospective single-centre chart review; the estimate depends on documentation of screening in records. Baseline and post-implementation periods were not randomized. |
| Thompson *et al*., 2019  (5) | Yes | Yes | Unclear | Yes | Yes | Yes | Yes | Yes | Yes | Prospective audit of 50 consecutive patients, with complete timing data for 46. Single community rehabilitation service limits generalizability; four patients lacked complete data. |
| Yan *et al*., 2025  (6) | Yes | Yes | Yes | Yes | Yes | Yes* | Yes | Yes | Yes | Multisite objective measurement is a strength; however, adherence was based on one monitored physical-therapy session at rehabilitation midpoint, so it should not be interpreted as episode-wide adherence. |

Response options: Yes = criterion adequately met; No = criterion not adequately met; Unclear = insufficient information to determine; N/A = not applicable.

* For Yan et al., the measurement was valid for determining whether the aerobic-intensity target was achieved during the monitored session; it does not establish adherence throughout the entire rehabilitation episode.

**References**

1.	M SA, Kooven S, Al-Mudahka N. Adherence of physical therapy with clinical practice guidelines for the rehabilitation of stroke in an active inpatient setting. Disabil Rehabil. 2019;41(15):1855–62.

2.	Auger LP, Filiatrault J, Allegue DR, Thomas A, Vachon B, Morales E, et al. Sexuality after a stroke: co-designing and pilot testing an evidence-based knowledge translation intervention to improve rehabilitation services. Disabil Rehabil. 2025;47(26):6898–908.

3.	McClure JA, Salter K, Foley N, Mahon H, Teasell R. Adherence to Canadian Best Practice Recommendations for Stroke Care: vascular cognitive impairment screening and assessment practices in an Ontario inpatient stroke rehabilitation facility. Top Stroke Rehabil. 2012;19(2):141–8.

4.	MacKenzie HM, Rice D, Teasell R, Macaluso S. Screening Adherence for Depression Post Stroke: Evaluation of Outpatients, a London Experience (SAD PEOPLE). Top Stroke Rehabil. 2019;26(1):6–17.

5.	Thompson S, Ranta A, Porter K, Bondi N. How much rehabilitation are our patients with stroke receiving? N Z Med J. 2019;132(1499):49–55.

6.	Yan Y, Eng JJ, Hung SH, Bayley MT, Best KL, Connell LA, et al. Aerobic minutes and step number remain low in inpatient stroke rehabilitation. PLoS One. 2025;20(7):e0328930.
