# Supplementary material for: Delivery of selected guideline-based care processes in adult stroke rehabilitation: a systematic review and meta-analysis
Source: Front Med (Lausanne). 2026 Oct 27;13:1963143. doi: 10.3389/fmed.2026.1963143 (PMC13747950; doi:10.3389/fmed.2026.1963143)
Supplement: Supplementary file 4 [file Table4.docx]

**Supplementary File 4**. Characteristics of guideline/recommendation sources and operationalization of included adherence outcomes.

| **Included study** | **Source guideline / recommendation** | **Year / version** | **Strength / grade** | **Recommendation wording relevant to pooled outcome** | **Applicable population** | **Type of measure** | **How adherence was operationalized** |
| --- | --- | --- | --- | --- | --- | --- | --- |
| **Ajimsha et al., 2019** | Physical Therapy After Acute Stroke (PAAS) guideline, developed within Hamad Medical Corporation, Qatar | Developed 2014-2015; specific formal version not reported in the study | Not reported | The PAAS guideline covered physiotherapy assessment, goal setting, management, and outcome measurement after acute stroke; a single recommendation corresponding to the pooled outcome was not specified. | Adult patients with ischemic stroke undergoing active inpatient rehabilitation and meeting study/chart eligibility criteria | Composite guideline-adherence measure | Overall adherence was calculated across the applicable guideline elements; the study reported 90/127 charts as meeting the operationalized overall adherence outcome (70.9%). |
| **Auger et al., 2025** | Evidence-based stroke sexual-rehabilitation recommendations incorporated into a knowledge-translation intervention | Intervention reported in 2025; no single guideline version or recommendation grade was specified for the pooled baseline outcome | Not reported | Stroke rehabilitation services should address sexuality/sexual health as part of rehabilitation; the publication operationalized this as provision of a sexuality-related rehabilitation service. | Individuals receiving inpatient stroke rehabilitation; service provision depended on individual rehabilitation context | Guideline-concordant rehabilitation care process | Monthly patient audits determined whether sexuality-related rehabilitation services had been provided. For the meta-analysis, the pre-intervention baseline value was used: 5/15 patients (33.3%). |
| **McClure et al., 2012** | Canadian Best Practice Recommendations for Stroke Care; vascular cognitive impairment recommendations | 2008 update / recommendations applicable at the time of the study | Cognitive screening recommendations included Evidence Levels B–C depending on the specific component | Patients with stroke at risk of cognitive impairment should undergo cognitive screening, with additional assessment where impairment is identified. | Stroke patients undergoing inpatient rehabilitation; applicability depended partly on ability to complete formal cognitive screening | Guideline recommendation | Charts were reviewed for documented cognitive screening during inpatient rehabilitation. The pooled outcome was 102/123 patients screened (82.9%). |
| **MacKenzie et al., 2019** | Canadian Best Practice Recommendations for Stroke Care; post-stroke depression screening recommendations | Recommendations current at study implementation; the 2015 Mood, Cognition and Fatigue update explicitly supported routine screening | Screening all stroke patients: Evidence Level B; timing across transition points: generally Evidence Level C | All stroke patients should be screened for depressive symptoms using a validated screening tool, with screening considered throughout the continuum of care. | Adult stroke survivors attending the outpatient stroke rehabilitation clinic | Guideline recommendation / screening process | Retrospective charts were reviewed for completion of standardized post-stroke depression screening. The primary meta-analysis used the routine-care pre-intervention period: 0/70 charts with standardized screening. |
| **Thompson et al., 2019** | New Zealand Stroke Network community rehabilitation recommendations; Ministry of Health community rehabilitation indicator | Recommendations/indicator current in 2018-2019 | Formal evidence grade not reported in the study | Community rehabilitation should commence promptly after hospital discharge; the Ministry indicator specified face-to-face contact within seven calendar days. | Patients with new stroke referred to the Wellington community rehabilitation service | Service quality indicator / recommended care-timing target | Time from hospital discharge to first face-to-face community rehabilitation contact was recorded prospectively. Adherence was defined as contact within seven calendar days; 17/46 patients (37.0%) met the target |
| **Yan et al., 2025** | AHA/ASA physical-activity recommendations and AEROBICS 2019 update | 2014 recommendation; updated 2020 | The paper did not operationalize the pooled endpoint according to a formal recommendation grade | Aerobic exercise after stroke is recommended at approximately 40–70% heart-rate reserve, 3–5 times/week, for 20–60 minutes/session. | Medically stable patients undergoing inpatient stroke rehabilitation who could take >5 steps with no more than one-person assistance | Guideline-derived therapy-intensity target | Aerobic minutes were objectively measured during one midpoint physical-therapy session. Meeting the target was defined as ≥20 minutes above 40% heart-rate reserve; 30/166 patients (18.1%) met this session-level target |
